# Intraductal papillary mucinous neoplasm originating from a heterotopic pancreas within the stomach

**DOI:** 10.1097/eus.0000000000000089

**Published:** 2024-11-08

**Authors:** Muyun Liu, Wei An, Jie Gao, Xingang Shi

**Affiliations:** 1 Department of Gastroenterology, Changhai Clinical Research Unit, Changhai Hospital, Shanghai 200433, China; 2 Department of Gastroenterology, No. 905 Hospital of PLA Navy Affiliated to Naval Medical University, Shanghai 200050, China.

**Figure 1:**
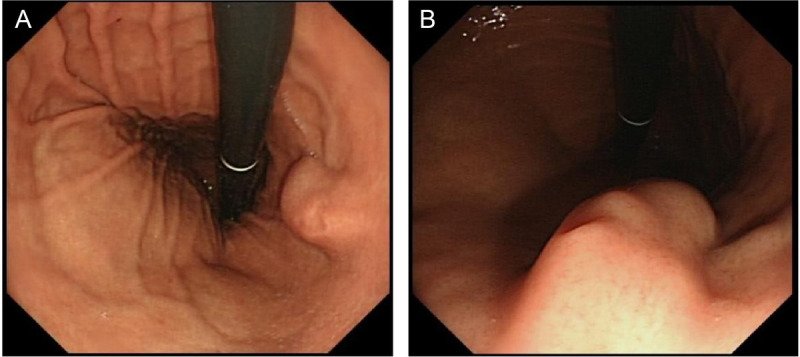
A, White-light gastroscopic examination showed a hemispherical elevation measuring approximately 1.0 cm × 2.0 cm on the posterior wall of the gastric body. B, Upon retracting the endoscope, a central depression in the lesion can be observed.

**Figure 2:**
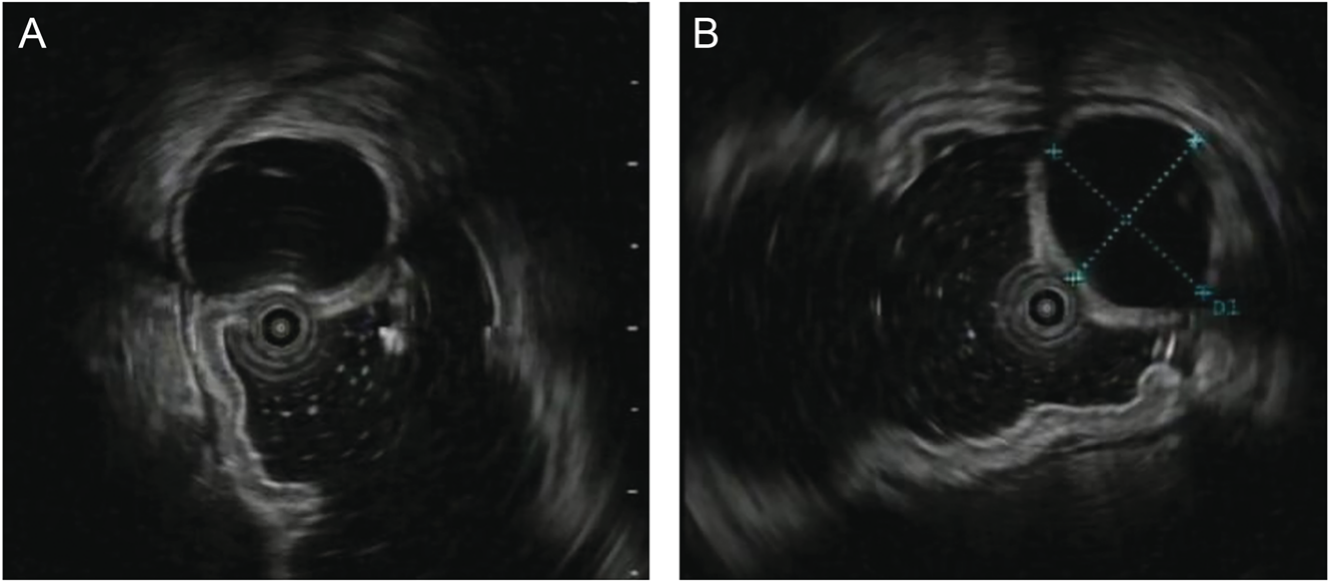
A, EUS revealed a hypoechoic mass exhibiting an elliptical shape and protruding both intraluminally and extraluminally. The lesion had well-defined borders and originated from the submucosal layer. The internal echotexture was heterogeneous, with areas of intermediate to high echogenicity, and included anechoic regions resembling cystic spaces. B, The mass is sized 1.3 cm × 1.2 cm under EUS measurement.

**Figure 3:**
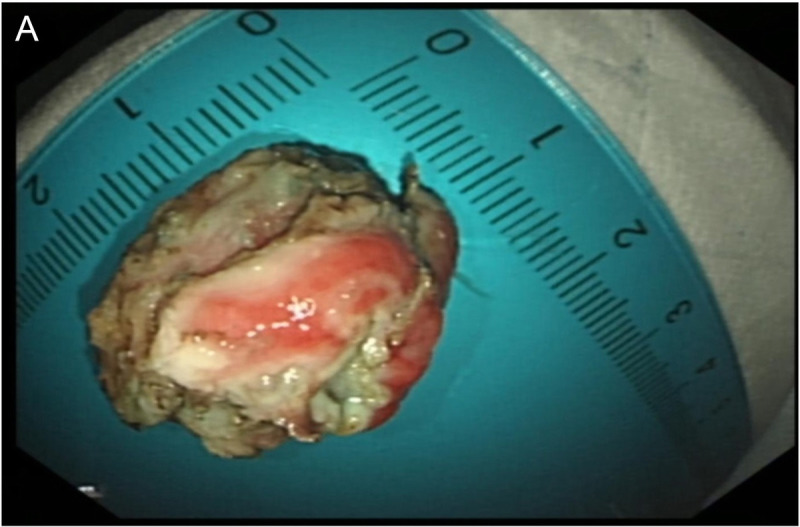
A, The solid tumor was resected via endoscopic submucosal dissection (ESD). The lesion exhibited a relatively firm texture and measured 2 cm × 1.5 cm × 1.5 cm.

**Figure 4:**
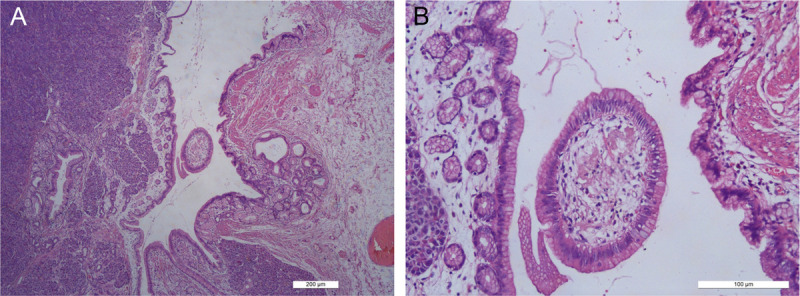
A, Post-ESD histology shows ectopic pancreatic tissue with dilated ducts, containing papillary-like structures (H&E, ×40 magnification). B, The papillary structures exhibit polypoid growth, with epithelial cells resembling those of gastric foveolar epithelium. The nuclei are basally located, and cellular atypia is minimal. A significant amount of mucin is present within the cells, suggesting a gastric phenotype (H&E, ×200 magnification).

A 32-year-old female patient was admitted due to the detection of a submucosal protrusion within the stomach in routine CT scan. The patient was asymptomatic without other history. Further gastroscopic examination showed that a hemispherical elevation measuring approximately 1.0 cm × 2.0 cm was identified on the posterior wall of the gastric body, with a visible depression on the surface under white-light observation [Figure 1A, B]. EUS revealed an oval mass sized 1.3 cm × 1.2 cm, protruding into and out of gastric lumen with clear boundaries, originating from the submucosal layer, with heterogeneous internal echoes, and local areas of medium to high echogenicity, including anechoic cystic spaces [Figure 2A, B]. Endoscopic submucosal dissection (ESD) was performed for diagnostic and therapeutic purposes. A tough nodular tumor measuring 2 cm × 1.5 cm × 1.5 cm was excised [Figure 3A]. Pathology revealed a gray-white, solid mass of medium consistency, and the pathological diagnosis was ectopic pancreas with intraductal papillary mucinous neoplasm (IPMN) (gastric type) formation accompanied with mild atypical hyperplasia [Figure 4A, B]. The patient experienced no discomfort postoperatively and discharged soon.

Ectopic pancreas typically presents as hypoechoic, isoechoic, or mixed echoic lesions under EUS. It can occur in any layer of the gastrointestinal wall, but most commonly in the submucosa, and can grow transmurally. The presence of duct-like structures is highly indicative for diagnosis.^[1]^ IPMN is a cystic lesion with malignant potential.^[2]^ Cases of ectopic pancreas complicated by IPMN are rare and only a few cases reported internationally.^[3–10]^ Among these cases, 3 developed malignancy. Therefore, early diagnosis is crucial for a favorable prognosis in such patients. Studies show that EUS has a sensitivity and specificity of 64% and 80%, respectively, for differentiating benign from malignant tumors, and it is superior to CT and MRI for lesions <2 cm in diameter.^[11]^ In our case, preoperative diagnosis of ectopic pancreas was challenging, and EUS-FNA offered a unique advantage.^[11]^ However, given the patient's young age, the patient opted for resection. Therefore, ESD was performed to remove the lesion following the guideline for the treatment of submucosal tumors (SMTs) within the gastrointestinal tract and revealed a favorable prognosis.
